# Accelerometer data reduction in adolescents: effects on sample retention and bias

**DOI:** 10.1186/1479-5868-10-140

**Published:** 2013-12-23

**Authors:** Mette Toftager, Peter Lund Kristensen, Melody Oliver, Scott Duncan, Lars Breum Christiansen, Eleanor Boyle, Jan Christian Brønd, Jens Troelsen

**Affiliations:** 1 Institute of Sports Science and Clinical Biomechanics, University of Southern Denmark, Odense, Denmark; 2 Centre for Intervention Research in Health Promotion and Disease Prevention, National Institute of Public Health, University of Southern Denmark, Copenhagen, Denmark; 3 Human Potential Centre, Auckland University of Technology, Auckland, New Zealand; 4 Dalla Lana School of Public Health, University of Toronto, Toronto, ON, Canada

**Keywords:** Physical activity, Measurements, Adolescents, Overweight, Accelerometer, Bias

## Abstract

**Background:**

Accelerometry is increasingly being recognized as an accurate and reliable method to assess free-living physical activity (PA) in children and adolescents. However, accelerometer data reduction criteria remain inconsistent, and the consequences of excluding participants in for example intervention studies are not well described. In this study, we investigated how different data reduction criteria changed the composition of the adolescent population retained in accelerometer data analysis.

**Methods:**

Accelerometer data (Actigraph GT3X), anthropometric measures and survey data were obtained from 1348 adolescents aged 11–14 years enrolled in the Danish *SPACE for physical activity* study. Accelerometer data were analysed using different settings for each of the three key data reduction criteria: (1) *number of valid days*; (2) *daily wear time*; and (3) *non-wear time*. The effects of the selected setting on sample retention and PA counts were investigated and compared. Ordinal logistic regression and multilevel mixed-effect linear regression models were used to analyse the impact of differing non-wear time definitions in different subgroups defined by body mass index, age, sex, and self-reported PA and sedentary levels.

**Results:**

Increasing the minimum requirements for daily wear time and the number of valid days and applying shorter non-wear definitions, resulted in fewer adolescents retained in the dataset. Moreover, the different settings for non-wear time significantly influenced which participants would be retained in the accelerometer data analyses. Adolescents with a higher BMI (OR:0.93, CI:0.87-0.98, p=0.015) and older adolescents (OR:0.68, CI:0.49-0.95, p=0.025) were more likely to be excluded from analysis using 10 minutes of non-wear compared to longer non-wear time periods. Overweight and older adolescents accumulated more daily non-wear time if the non-wear time setting was short, and the relative difference between groups changed depending on the non-wear setting. Overweight and older adolescents did also accumulate more sedentary time, but this was not significant correlated to the non-wear setting used.

**Conclusions:**

Even small differences in accelerometer data reduction criteria can have substantial impact on sample size and PA and sedentary outcomes. This study highlighted the risk of introducing bias with more overweight and older adolescents excluded from the analysis when using short non-wear time definitions.

## Background

Within the last decade, the use of objective measures to quantify physical activity (PA) and sedentary behaviour in population-based studies of children and adolescents has increased. Accelerometry is regarded a valid and reliable method to quantify frequency, duration and intensity of free-living PA and has become increasingly prevalent in surveillance, observational, and intervention research in children and adolescent [1-3]. Nonetheless, the use of accelerometers comes with a number of challenges. Although data reduction issues have received increased attention in recent years [4,5], a consensus on a standard data reduction protocol remains elusive. A recent literature review of 183 studies published between 2005 and 2010 indicated a continuing lack of agreement amongst researchers on protocols used to collect, process, and score accelerometer data from children and adolescents [6]. Some elements of accelerometer data reduction have been repeatedly studied, whereas others have not received the same attention.

Previous studies have investigated the effects of differing accelerometer epoch durations [2,7], monitor placement [8,9], count thresholds for defining PA intensity and sedentary behaviour [6,10,11], monitor types [12-15], and data inclusion criteria such as number of monitoring hours per day and number of days [4,5,16], on PA measurement in children and adolescents. Studies have used between six and over 12 hours per day to define a valid day with eight to ten hours per day being the most commonly used [6]. With regards to number of days, four or five days of monitoring have been recommended as an adequate number of monitoring days [17]; however, some studies have used a single day as a minimum criterion e.g. [18-20], and others have used up to ten days, e.g. [21]. This indicates that no consensus has been reached on the minimum number of days required to gain an accurate picture of adolescent PA levels [6].

Currently, no standards exist concerning the optimal approach to deal with non-wear time, that is events when the accelerometer was removed, and studies in children and youth have used non-wear periods that range between ten and 180 minutes of consecutive zero counts [6,12-15]. A potential problem regarding different definitions of non-wear time is that it is difficult to determine the difference between sedentary behaviour and true non-wear (in both cases, the accelerometer may register zero counts). Therefore, the quantification of non-wear time may also influence sample retention and have significant effects on PA and sedentary behaviour quantification [4,6,22]. In particular, considerable over- or under-estimation of time spent sedentary may occur as a result of too long or too short non-wear time setting, respectively. If the non-wear setting is too long, there is a risk of over estimating time spent sedentary, because maybe the accelerometer was actually not worn. On the other hand, if the non-wear setting is too short, true sedentary time would be categorised as non-wear and thus excluded from analysis. Consequently, it is possible that an arbitrary definition of non-wear time may bias results for populations especially, those who are highly sedentary. Because those who are highly sedentary are more likely to accumulate long/many strings of consecutive zeroes, and thus depending on the non-wear criterion used, there is a risk of classifying sedentary time as non-wear time. To date, no research has to our knowledge investigated these issues in free-living youth populations.

Using accelerometer data derived from a large sample of adolescents, the aims of this study were to: 1) investigate the impact of different inclusion criteria for number of valid days, daily wear time and non-wear time definitions on sample retention and PA counts; and 2) investigate if differing non-wear time definitions introduced any bias to the sample with regards to adolescents’ characteristics (age, sex, BMI and self–reported PA level and sedentary time).

## Methods

### Study design

We used baseline data from the Danish *SPACE for physical activity* study, a multicomponent school-based intervention study aimed at improving PA levels among adolescents. The primary outcome measure in the SPACE study was PA as measured by accelerometer. Fifth and sixth graders (aged 11–14 years) were recruited from 14 schools and baseline data collection was conducted in April-June 2010. A total of 1348 adolescents (48.6% female) were enrolled in the baseline study, and 1296 provided accelerometer data. The reasons for missing data before the data reduction analyses were mainly due to adolescents not willing to participate in the accelerometer study or adolescents being absent from school in the period of data collection. Furthermore, a few monitors were broken when adolescents returned them and some were lost during the data collection period. For more detailed information about the SPACE study and the study design see the study protocol [23].

### Ethical approval

Personalised information about the study was distributed to parents and students. Parents of the participating adolescents received a passive informed consent form that explained the nature and procedures of the study. It was possible to withdraw at any stage of the study. The Danish National Committee on Health Research Ethics reviewed the study protocol and concluded that formal ethical approval was not required. The study was registered and listed in the Danish Data Protection Agency (reference number: 2009-41-3628).

#### Accelerometer

Objective PA and sedentary behaviour were obtained using the Actigraph GT3X Activity Monitor. Previous studies examining methodological issues have mainly used earlier monitors, although these monitors are compatible with the current model that was used in this study [24]. All adolescents who participated in the project were asked to wear an accelerometer for five weekdays and two weekend days, but due to holidays/long weekends some adolescents wore the accelerometer for eight days. The accelerometer was attached to an elastic belt which the adolescents were instructed to wear around their waists continuously throughout the day except when showering/water activities and sleeping. The accelerometer data were recorded at two-second epochs, with total PA expressed as counts per minute (cpm), the vertical axis was used and sedentary time was defined as activity ≤100 cpm [25]. The normal filter option was used.

Verbal and written instructions by the research staff or by trained assistants were given to the adolescents regarding how to wear the accelerometer. To increase compliance in wearing the accelerometer, the adolescent or one of their parents were offered a free text message reminder on their mobile phone every morning.

#### Adolescent questionnaires

The self-completed adolescent questionnaire contained among other things questions on their PA and their sedentary behaviour in leisure time. Majority of questions came from previously validated children and adolescents’ health surveys such as the Health Behaviour in School-aged Children [26]. The students completed the questionnaire on a computer during one school lesson (45 minutes) within the week that they wore their accelerometer. A teacher was present during the session and instructed the adolescents how to get started and helped if there were any questions or problems with comprehension. All adolescents were instructed to fill out the questionnaire individually, without talking to classmates [23]. The adolescents were asked to estimate their PA level in leisure time during a typical week. The activity levels were as follows: vigorous (performs sports several times a week), moderate (doing sport approx. once a week, and besides that physical active every day, cycling, walking, playing), light (enjoy being physical active, but do not attend any sport clubs, cycle, walk and physical active when playing) and sedentary (prefer to watch TV, play video games, listen to music or other sedentary activities). The adolescents were furthermore asked to estimate the amount of time they spent on three different sedentary behaviours (watching TV; computer work; doing homework and/or reading) in leisure time during a typical week for weekdays and for weekends. Time spent was measured on a six-level ordinal scale of none, one, two, three, four, or five or more hours per day for each of the activities). For the analyses we dichotomised the items on sedentary behaviour into an aggregated measure of below/above 4 hours daily sedentary behaviour during weekdays and weekend days [26].

#### Anthropometry

Height and weight were assessed by standard anthropometric procedures. Height was measured to the nearest millimetre using a portable stadiometer (SECA Leicester portable Height Measure). Weight was assessed to the nearest decigram using a medical scale (Tanita BWB-800S Digital Scale). Weight status was classified as underweight/normal weight or overweight/obese according to the international cut-off point for overweight proposed by Cole et al [27].

#### Age and sex

The child’s age and sex were obtained from school records using the Danish Civil Registration System [28,29], which is a registry of all socio-demographic characteristics of every resident in Denmark.

#### Accelerometer data treatment and statistical analyses

Descriptive statistics were used to describe the study population with regards to sex, age, BMI, self-report PA and self-report sedentary time (Table 1).

**Table 1:** Characteristics of the study population, N = 1348

	**Girls**	**Boys**
**Sex**, % (n = 1348)	48.4	51.6
**Age**, mean y (SD) (n = 1348)	12.4 (0.62)	12.5 (0.63)
**BMI**, mean kg/m^2^ (SD) (n = 1266)	18.9 (3.04)	18.8 (2.99)
**Weight status** (BMI thresholds)* (n = 1266)		
*Under-/normal weight*	85.5	85.5
*Overweight/obese*	14.6	14.5
**Self-reported PA in leisure time **% (n = 1313)**	638 (48.6)	675 (51.4)
*Performs sports several times a week. Hard exercise*	37.2	53.3
*Doing sport approx. once a week, and besides that physical active every day, cycling, walking, playing*	35.3	19.4
*Enjoy being physical active, but do not attend any sport clubs. Cycle, walk and are physical active when playing*	24.1	19.1
*Prefer to watch TV, play video games, listening to music or other sedentary behaviour*	3.5	8.2
**Self-reported sedentary leisure time** (n = 1313)**		
*4 + hours daily sedentary time (watching TV, playing computer games, reading etc.) in leisure time in weekdays and weekend days, %*	58.2	67.2

*Age and sex standardized [27,28].

**Significant differences between girls and boys (p < 0.05).

N varies due to different data sources.

Accelerometer data were analysed using different settings for the three key data reduction criteria: 1) *number of valid days* (1, 2, 3, 4, 5, 6 and 7–8 days), 2) *daily wear time* (6, 8, 9, 10 and 12 hours per day) and 3) *non-wear time* (10, 20, 30, 60 and 90 minutes of consecutive zeroes). These categories were chosen based on data reduction criteria often used in other studies [1,6,8]. The following parameters in the data reduction analyses were fixed: 30 second epochs, maximum physiologically allowed value of 20,000 cpm. [5] and two non-zero count epochs permitted in blocks of non-wear [30]. Based on the different combinations of data reduction settings, participant retention and PA level (mean cpm) was calculated (Table 2). The software program Propero Actigraph Data Analyzer version 18 (University of Southern Denmark, Odense, Denmark) was used for processing accelerometer data.

**Table 2:** Adolescents included in analysis in different data reduction criteria: Hours to define a valid day, number of valid days and non-wear time), N = 1296

	**1 day**			**2 days**			**3 days**			**4 days**			**5 days**			**6 days**			**7-8 days**			
***Hours***	***N***	***(%)***	***PA, cpm***	***N***	***(%)***	***PA, cpm***	***N***	***(%)***	***PA, cpm***	***N***	***(%)***	***PA, cpm***	***N***	***(%)***	***PA, cpm***	***N***	***(%)***	***PA, cpm***	***N***	***(%)***	***PA, cpm***	
***6***	1288	(99.4)	560.0	1282	(98.9)	560.7	1265	(97.6)	561.2	1249	(96.4)	561.5	1217	(93.9)	561.6	1157	(89.3)	558.9	1049	(80.9)	562.8	
***8***	1284	(99.1)	563.7	1275	(98.4)	564.2	1256	(96.9)	564.5	1234	(95.2)	565.0	1184	(91.4)	565.4	1103	(85.1)	563.3	947	(73.1)	567.9	
***9***	1282	(98.9)	566.3	1270	(98.0)	566.9	1251	(96.5)	567.0	1225	(94.5)	567.1	1171	(90.4)	567.5	1063	(82.0)	564.3	869	(67.1)	572.8	***90 min non-wear***
***10***	1281	(98.8)	569.3	1259	(97.1)	569.6	1239	(95.6)	570.1	1211	(93.4)	568.2	1134	(87.5)	569.8	999	(77.1)	569.4	772	(59.6)	572.7	
***12***	1267	(97.8)	572.3	1241	(95.8)	573.6	1205	(93.0)	571.0	1133	(87.4)	571.6	978	(75.5)	571.4	710	(54.8)	573.4	360	(27.8)	569.9	
***6***	1286	(99.2)	571.5	1279	(98.7)	572.3	1263	(97.5)	572.0	1247	(96.2)	571.5	1212	(93.5)	572.2	1150	(88.7)	567.2	1024	(79.0)	570.5	
***8***	1281	(98.8)	575.4	1273	(98.2)	575.2	1253	(96.7)	575.5	1231	(95.0)	575.4	1177	(90.8)	573.5	1087	(83.9)	572.2	921	(71.1)	575.6	
***9***	1279	(98.7)	577.1	1264	(97.5)	577.1	1247	(96.2)	577.3	1219	(94.1)	575.9	1159	(89.4)	574.8	1040	(80.2)	573.9	839	(64.7)	577.7	***60 min non-wear***
***10***	1277	(98.5)	579,1	1256	(96.9)	579.3	1234	(95.2)	578.6	1207	(93.1)	577.1	1128	(87.0)	576.3	980	(75.6)	575.9	741	(57.2)	575.4	
***12***	1263	(97.5)	580.1	1237	(95.4)	580.4	1191	(91.9)	577.5	1111	(85.7)	576.3	937	(72.3)	575.9	653	(50.4)	575.4	313	(24.2)	563.7	
***6***	1284	(99.1)	589.0	1277	(98.5)	589.1	1261	(97.3)	588.0	1244	(96.0)	587.6	1201	(92.7)	587.0	1137	(87.7)	582.7	998	(77.0)	581.5	
***8***	1278	(98.6)	591.3	1268	(97.8)	590.8	1249	(96.4)	590.5	1227	(94.7)	590.5	1169	(90.2)	588.4	1070	(82.6)	585.3	888	(68.5)	586.0	
***9***	1277	(98.5)	592.2	1260	(97.2)	592.1	1242	(95.8)	591.8	1213	(93.6)	590.8	1146	(88.4)	589.9	1016	(78.4)	585.6	811	(62.6)	587.2	***30 min non-wear***
***10***	1275	(98.4)	593.8	1252	(96.6)	593.4	1229	(94.8)	591.8	1192	(92.0)	591.1	1106	(85.3)	589.9	945	(72.9)	586.4	691	(53.3)	583.9	
***12***	1256	(96.9)	594.9	1230	(94.9)	593.6	1181	(91.1)	591.1	1070	(82.6)	589.7	872	(67.3)	587.6	569	(43.9)	576.0	238	(18.4)	569.3	
***6***	1282	(98.9)	600.6	1277	(98.5)	600.6	1259	(97.1)	599.5	1244	(96.0)	599.0	1199	(92.5)	598.2	1128	(87.0)	594.3	981	(75.7)	593.4	
***8***	1277	(98.5)	602.7	1265	(97.6)	601.5	1248	(96.3)	601.7	1224	(94.4)	601.2	1166	(90.0)	599.8	1058	(81.6)	596.7	870	(67.1)	596.3	
***9***	1276	(98.5)	604.2	1257	(97.0)	603.8	1239	(95.6)	603.4	1210	(93.4)	602.4	1137	(87.7)	601.1	996	(76.9)	598.8	772	(59.6)	600.6	***20 min non-wear***
***10***	1269	(97.9)	595.5	1252	(96.6)	595.1	1227	(94.7)	594.7	1186	(91.5)	593.0	1091	(84.2)	593.5	919	(70.9)	591.2	630	(48.6)	592.3	
***12***	1254	(96.8)	605.7	1222	(94.3)	604.2	1167	(90.0)	603.3	1036	(79.9)	603.3	806	(62.2)	596.7	502	(38.7)	592.6	182	(14.0)	591.6	
***6***	1281	(98.8)	633.2	1275	(98.4)	632.9	1257	(97.0)	631.1	1242	(95.8)	632.0	1194	(92.1)	630.4	1120	(86.4)	626.5	955	(73.7)	627.3	
***8***	1275	(98.4)	636.5	1261	(97.3)	634.8	1244	(96.0)	634.9	1214	(93.7)	634.2	1150	(88.7)	632.6	1025	(79.1)	630.5	816	(63.0)	634.5	
***9***	1270	(98.0)	637.2	1253	(96.7)	635.7	1231	(95.0)	635.5	1193	(92.1)	633.5	1103	(85.1)	634.1	947	(73.1)	634.7	682	(52.6)	640.1	***10 min non-wear***
***10***	1267	(97.8)	638.6	1244	(96.0)	638.5	1211	(93.4)	636.1	1154	(89.0)	637.0	1035	(79.9)	641.0	819	(63.2)	638.7	473	(36.5)	639.8	
***12***	1224	(94.4)	641.4	1179	(91.0)	643.4	1070	(82.6)	647.1	884	(68.2)	645.9	597	(46.1)	642.9	286	(22.1)	647.7	85	(6.6)	667.7	

Data reduction criteria used: 30 sec epoch, 24 hour duration, allow 2 activity epochs in blocks of non-wear.

Presented as Number (%) and with the corresponding average physical activity (PA) counts (mean counts per minutes).

When investigating the effect of using different non-wear criteria it was necessary to fix the other two data reduction criteria: *number of valid days* and *daily wear time* in order to minimise complexity. Adolescents with at least five days of ten hours each were defined as valid, based on commonly used and recommended setting in other studies [6,17]. Differences in characteristics between adolescents with and without sufficient valid data were analysed using Wilcoxon rank-sum (Mann–Whitney) for continuous variables (age, BMI), and chi-squared tests for categorical variables (sex, age groups, weight status, self-reported PA and self-reported sedentary time) (Table 3).

**Table 3:** Adolescents characteristic with no accelerometer data compared to adolescents with valid accelerometer data, n = 1337 (Initial N = 1296)

	No accelerometer data*	Valid accelerometer data**	**p-value**
**Sex**, % (n = 1337)	(n = 205)	(n = 1132)	0.011
Girls	40.5	50.1	
Boys	59.5	49.9	
**Age**, mean year (n = 1337)	12.7 (n = 205)	12.6 (n = 1132)	0.003
**Age groups**	(n = 205)	(n = 1132)	0.030
*11-12 years*	65.4	72.8	
*13-14 years*	34.6	27.2	
**BMI**, mean kg/m2 (n = 1266)	19.3 (n = 180)	18.8 (n = 1086)	0.016
**Weight status** (BMI thresholds)*** (n = 1266):	(n = 180)	(n = 1086)	0.200
*Under-/normal weight*	82.7	86.3	
*Overweight/obese*	17.3	13.7	
**Self-reported PA in leisure time**, % (n = 1313):	(n = 194)	(n = 1119)	0.042
*- Performs sports several times a week (vigorous activity)*	47.4	45.1	
*- Doing sport approx. once a week, and besides that physical active every day, cycling, walking, playing (moderate activity)*	19.6	28.4	
*- Enjoy being physical active, but do not attend any sport clubs. Cycle, walk and are physical active when playing (light activity)*	24.7	21.0	
*- Prefer to watch TV, play video games, listening to music or other sedentary behaviour (sedentary)*	8.3	5.5	
**Self-reported sedentary leisure time**, %: 4 h + of daily sedentary time (TV, computer, reading) in weekdays and weekend days (n = 1313)	66.0 (n = 194)	62.3 (n = 1119)	0.326

N varies due to different data sources.

Number of valid days and daily wear time were fixed to 5 days of 10 hours in defining valid data.

*No valid data in any of the non-wear settings. **Valid data when using the 10, 20, 30 or 60/90 minutes of non-wear criterion. ***Age and sex standardized [27].

To examine if different non-wear time definitions introduced any bias to the sample with regards to adolescents’ characteristics (age, sex, BMI, self–reported PA level and self-reported sedentary time), we combined five datasets of varying non-wear time definitions (10, 20, 30, 60 and 90 minutes of consecutive zeroes). Based on this new dataset we created a non-wear time ordinal variable with six categories (0–6) defined by whether or not the adolescent would be included in the analysis based on the length of time of non-wear settings (category 0 = no valid accelerometer data, category 1 = included in analysis when using a 90 minutes non-wear criterion, category 2 = included when using a 60 minutes non-wear criterion etc. to category 6 = included in the 10 minute non-wear category). We conducted univariate ordinal logistic regressions with the outcome being included/excluded from the analysis for each of the five non-wear criteria, for each of the explanatory variables: age, sex, BMI, self–reported PA level and self-reported sedentary time in the model (Table 4).

**Table 4:** Ordinal logistic regression model with the outcome being in/out in the analysis in the different non-wear settings, n = 1132 (Initial N = 1296)

	**OR**	**95% CI**	**p-value**
**BMI** (kg/m2)	0.93	0.87-0.98	0.015
**Age** (in years)	0.68	0.49-0.95	0.025
**Sex** (boys)	1.21	0.80-1.82	0.374
**Self-reported PA in leisure time**;			
Vigorous activity	1(ref)		
Moderate activity	1.60	0.93- 2.74	0.090
Light activity	1.08	0.63-1.85	0.777
Sedentary behaviour	0.83	0.36-1.92	0.665
**Self-reported 4 h + of daily sedentary leisure time**	0.91	0.59-1.40	0.658

Note: Variables included in model one at a time. (Running the full model with all variables included at the same time does not change effect sizes).

Number of valid days and daily wear time were fixed to at least 5 days of 10 hours.

OR, 95% CI.

To further explore the effect of using different definitions of non-wear time, we investigated the length of all daily non-wear periods i.e., the total number of daily minutes that was excluded from the analyses in the different non-wear settings for each different subgroup (i.e. for age, sex, BMI, self-reported PA level and self-reported sedentary time). Daytime was defined as the period from 6 am to 10 pm. We used a multilevel mixed effect linear regression model with interaction terms to examine the effect of the different non-wear time criteria on each of the variables: age group, sex, weight status, self-reported PA and self-reported sedentary time on non-wear time. Respondent ID was included as a random effect with repeated measures for each subject as level one and the subject as level two. A significance level of 0.05 was used (Table 5).

**Table 5:** Non-wear time (daily minutes) and sedentary time (% of total time accepted, daily minutes) in the different non-wear criteria for weight status and age groups, n = 1087 (Initial N = 1296)

	**Non-wear time**								**Sedentary time**					
	***Weight status***				***Age group***				***Weight status***			***Age group***		
	***Under-/normal weight***	***Over-weight***	***Diff (%)***	***p-value***	***11-12 years***	***13-14 years***	***Diff (%)***	***p-value***	***Under-/normal weight***	***Over-weight***	***p-value***	***11-12 years***	***13-14 years***	***p-value***
**90 min non-wear**	15	16	1 (6.7)	0.551	14	17	3 (21.4)	0.131	59% (484 of 812)	62% (503 of 815)	0.001	59% (481 of 811)	62% (502 of 816)	<0.001
**60 min non-wear**	18	20	2 (11.1)	0.527	18	21	3 (16.7)	0.115	59% (478 of 809)	61% (497 of 812)	0.002	59% (475 of 808)	61% (497 of 812)	<0.001
**30 min non-wear**	27	29	2 (7.4)	0.364	26	31	5 (19.2)	0.010	58% (469 of 801)	61% (486 of 804)	0.005	58% (466 of 800)	61% (486 of 803)	<0.001
**20 min non-wear**	35	40	5 (14.3)	0.049	34	40	6 (17.6)	<0.001	58% (462 of 795)	60% (476 of 795)	0.006	58% (459 of 794)	60% (477 of 794)	<0.001
**10 min non-wear**	55	65	10 (18.2)	<0.001	54	64	10 (18.5)	<0.001	57% (442 of 778)	59% (451 of 773)	0.016	57% (439 of 778)	59% (453 of 774)	<0.001
	*Overall significant interaction* (p < 0.01)				*Overall significant interaction* (p < 0.01)				*Overall non-significant interaction* (p > 0.05)			*Overall non-significant interaction* (p > 0.05)		

Daytime activity included (defined as the period from 6 am to 10 pm). Sedentary time analyses: Proportion of sedentary time out of total time accepted in each of the non-wear settings. Number of valid days and daily wear time were fixed to at least 5 days of 10 hours. Results are based on a multilevel mixed effect linear regression model with interaction terms.

A potential problem regarding different definitions of non-wear time is that it is difficult to determine the difference between sedentary behaviour and true non-wear (in both cases, the accelerometer may register zero counts); consequently, we replicated the multilevel mixed effect analysis with interaction terms using sedentary time (accelerometer activity ≤100 cpm) as the outcome in order to determine any differences in sedentary time (proportion of total time accepted) by the different subgroups in the different non-wear settings (Table 5).

Finally, to further explore the impact of using different non-wear time settings, the software programme Propero Actigraph Data Analyzer was used to examine the daily number of non-wear periods that was removed from the data set in each of the non-wear criteria (Table 6).

**Table 6:** Distribution of the number of daily non-wear periods in the different non-wear settings, n = 1132 (Initial N = 1296)

**Non-wear criteria**	**Median**	**Mean**	**Variance**	**Maximum**
90 minutes of non-wear	0	0.1	0.11	2
60 minutes of non-wear	0	0.2	0.17	3
30 minutes of non-wear	0	0.4	0.48	6
20 minutes of non-wear	0	0.8	1.00	8
10 minutes of non-wear	2	2.5	5.02	17

Day time defined as the period from 6 am to 10 pm.

All statistical analyses were conducted using StataSE12.

## Results

A total of 1348 adolescents entered the study (48.4% female, mean age 12.5 years, 14.5% classified as overweight or obese) (Table 1). We found that 45.5% of the adolescents participated in organized sport several times a week and boys (53.3%) participated in more sports than girls (37.2%). Approximately, two thirds of the adolescents reported they spent at least four hours daily in sedentary time during weekdays and on the weekend and boys (67.2%) had more self-reported sedentary time than girls (58.2%) (Table 1).

### Daily wear time and number of valid days

Accelerometer data were obtained from a total of 1296 adolescents (prior to data reduction) out of the 1348 eligible adolescents. Table 2 shows the number of adolescents included in accelerometer analysis based on the different wear time settings and the corresponding PA level (mean cpm). Increasing the minimum requirements for daily wear time and the number of valid days substantially reduced the number of adolescents included in the study. For example, applying a commonly used criteria of eight hours/day, three days, and 20 non-wear minutes there were 1248 adolescents included; however, using criteria of ten hours/day, five days, and 20 non-wear minutes there were 1091 adolescents included which meant 157 adolescents were excluded (12% of original sample).

Based on a visual inspection of the data, overall average PA counts in general increased slightly when the number of hours to define a valid day went up. This trend was most distinct using the 10 and 90 minutes of non-wear criteria. Changing the number of valid days did not impact PA levels in a systematic way (Table 2).

### Non-wear time

Lengthening the non-wear time duration increased the number of adolescents included in the study (Table 2). For example if the criterion of 60 minutes non-wear time was used for the previous examples, only 125 adolescents (10%) were excluded instead of 157 using the 20 minutes of non-wear time criterion. With regards to PA outcome, the average activity level decreased with an increase in non-wear time duration. For instance, the average PA level decreased from 641 cpm when applying 10 minutes of non-wear time (10 hours/day, 5 days) to 570 cpm (10 hours/day, 5 days) using 90 minutes of non-wear (Table 2).

When the number of valid days and daily wear time were fixed to five days and ten hours, a total of 205 (15.3%) adolescents had insufficient accelerometer data for all of the non-wear settings (Table 3). Adolescents with insufficient accelerometer data had a significant higher BMI (p = 0.016) compared to respondents with valid accelerometer data. Furthermore, excluded adolescents were more likely to be boys (p = 0.011) and reporting light/no PA in leisure time (p = 0.042). Having more than four hours of daily self-reported sedentary time was not significantly related to having valid accelerometer data (p = 0.326).

The next step was to investigate whether characteristics differed among adolescents with valid accelerometer data using a 10, 20, 30, 60 or 90 minutes of non-wear time criterion. Because there were very few adolescents in the 90 minutes non-wear category, we collapsed the categories of 90 and 60 minutes of non-wear time together to improve the precision of the model.

The ordinal logistic regression analysis (Table 4) showed that with a one point increase in BMI, the odds ratio of being included in the PA analysis was 0.93 (CI:0.87-0.98, p = 0.015) going from one level of non-wear to another starting at the 60/90 minutes of non-wear time criterion and going “up” to the 10 minutes of non-wear. In other words, more adolescents were excluded with a higher BMI using 10 minutes of non-wear compared to the longer non-wear time periods. Increasing age was similarly associated with lower odds (OR:0.68, CI:0.49-0.95, p = 0.025) for being in the analysis in the shorter non-wear settings. There were no significant differences in the likelihood of being included in analysis using different non-wear definitions by sex, self-reported PA, or self-reported sedentary time in leisure time (Table 4).

Table 5 shows the distribution of daily minutes of non-wear time and the proportion of daily minutes of sedentary time for the overweight versus the normal-weight and for the younger (11–12 years) versus the older (13–14 years) adolescents in the different non-wear settings based on the interaction terms from the multilevel mixed effect linear regression models. The overweight adolescents had significantly more daily minutes of total non-wear time in the shorter non-wear settings (10 and 20 minutes of non-wear time) than the normal weight adolescents. Using 10 minutes as the non-wear setting, overweight adolescents’ accumulated 18% (10 minutes) more non-wear time compared to the normal weight adolescents, whereas there was no difference between normal weight and overweight adolescents in the 90 minute of non-wear criterion. The significant interaction term (p = 0.002) also confirms this pattern.

Including age in the model, as a categorical variable, also revealed significant differences between younger and older adolescents with the older adolescents accumulating more daily minutes of non-wear using the 10, 20 and 30 non-wear time definitions. The most distinct difference was for the 10 minutes of non-wear setting with the older adolescents accumulating 22% more non-wear time than the younger adolescent (Table 5).

From the analyses of time spent in sedentary behaviour for the different subgroups, the results showed, as expected, that the longer non-wear setting, the more sedentary time was accumulated. The overweight and older adolescents compared to the normal-weight had more sedentary time in every non-wear settings, and there was no overall significant interaction. In general the non-overweight and younger adolescents spent 57-59% of their recorded time in sedentary behaviour, whereas the corresponding estimates were 59-62% among the overweight and older adolescents (Table 5).

We repeated the analyses of non-wear and sedentary time for sex, self-reported PA, and sedentary time, and did not find any significant differences (data not shown).

Table 6 shows the results from the analysis of the number of daily non-wear periods. When comparing the number of non-wear periods (events when the accelerometer was removed) in the different non-wear time settings, it can be seen that using a 10 minute non-wear definition resulted in a median of 2 and a mean of 2.5 non-wear periods during the day (from 6 am to 10 pm), and the maximum number of non-wear periods during the day was as high as 17. Lengthening the definition of non-wear time duration in the analyses substantially reduced the number of non-wear periods, and using for example a 60 minute non-wear definition resulted in a median of 0, a mean of 0.2 and three maximum daily non-wear periods during the day.

## Discussion

### Retention and physical activity

The first aim in this study was to investigate the impact of differing inclusion criteria: number of valid days, daily wear time and non-wear time definitions on sample retention and PA. In a detailed analysis with the combination of data reduction criteria often used (1–7 days, 6–12 hours per/day, and non-wear periods of 10, 20, 30, 60 and 90 minutes), we found that lowering the minimum daily wear time criteria and the number of valid days and lengthening the non-wear time period resulted in a substantial increase in participant inclusion. Using longer non-wear definitions resulted in more participants, but with less activity. The latter is a logical consequence of stipulating that longer strings of consecutive zeroes could be retained. Other studies, e.g. Colley et al. and Masse et al. [4,5]. have found similar results; however, their reported analyses were not so detailed with the number of combinations used and their cohorts’ sample sizes were smaller. Thus, this study both replicates and extends their findings by including a wider range of data reduction criteria and a larger sample.

Based on a visual inspection, we found that PA levels increased slightly with increasing minimum requirements for hours to define a valid day. A possible explanation is that more physical active adolescents to a greater extent are motived to wear the accelerometer continuously during the day.

### Non-wear time

A recent review of 183 accelerometer studies concluded that non-wear setting was the criterion most often not reported, with missing information in 48.6% of the studies [6]. In the present study we highlighted the importance of reporting the non-wear setting by combining and analysing five datasets with varying non-wear time definitions, and illustrated the significant impact applying these criteria had on the data material, with regards to sample retention, and also more importantly because of the risk of introducing bias to the study sample.

We investigated if differing non-wear time definitions introduced any bias to the sample with regards to adolescents’ characteristics (age, sex, BMI and self–reported PA level and sedentary time). We found differences between adolescents with and without valid accelerometer data using any non-wear definition. Boys and younger adolescents were less likely to provide valid data, and adolescents with valid data had a lower BMI. This is in contrast to Van Coevering et al. [15], who found that overweight adolescents (grades 6–8) were more likely to provide seven days of complete data. A possible explanation for the difference could be that the authors used a 180 minute non-wear criterion whereas in this study we used between 10 minutes and 90 minutes. Another study by Mattock et al. [16] reported similar results to our study where adolescents with valid data tended to be girls, younger in age and with a lower BMI.

The analysis of using differing non-wear settings revealed a risk of excluding specific subgroups, namely the overweight and older adolescents, when using shorter non-wear criteria in the data reduction. Furthermore, based on a multilevel mixed effect linear regression model with interaction terms, we can conclude that among the adolescents not excluded from analyses, there was a tendency that the overweight and older adolescents accumulated more daily non-wear time in the short non-wear time settings, and the relative difference between groups changed depending on the non-wear setting.. For example, using 10 minutes as the non-wear setting, overweight adolescents’ accumulated 18% more non-wear time compared to the under-/normal weight adolescents, whereas using 60 minutes as the non-wear setting the difference between the two groups were 11%. Older adolescents accumulated significant more non-wear time compared to younger adolescents in all the non-wear settings. These findings raise some interesting questions. Do the overweight and older adolescents remove their accelerometer more often and for short periods? If you are overweight and not very interested in PA your compliance rate could be low, but would you then bother taking the accelerometer on and off several times during the day? It could also be that the sensitivity of the monitor to movement when worn by overweight participants is an issue, and there are for example studies e.g. [31,32] suggesting that pedometers are less accurate in counting steps in overweight and obese adolescents than normal weight individuals.

### True non-wear or sedentary time?

Another explanation for the differential effect could be that overweight and older adolescents spend more time in sedentary time, and therefore record more zero counts on the accelerometer, resulting in a risk of misclassifying sedentary time as non-wear time, when using shorter non-wear settings. We examined similarly the amount of daily sedentary time in the subgroups with regards to weight status and age group, and found that the overweight and older adolescents compared to the younger and normal weight adolescents accumulated more sedentary time in every non-wear setting, this was however not significantly associated with the non-wear setting used. Based on our findings, one could argue that overweight and older adolescents need a longer non-wear definition because of their sedentary behaviour records less movement. But then comparability between groups will always be questionable. Is it better to have less error in one group (i.e. overweight) but also less comparability? Therefore we will not suggest differential inclusion criteria by population sub group, as this makes data treatment very messy when it comes to including all participants in group analyses. Instead it might be that a longer non-wear definition is used and post-hoc subgroup analyses are considered in studies where sample size permits this.

In this study the Actigraph GT3X with the normal filter option was used. We acknowledge that the use of other accelerometer models and filters may not produce comparable results. Recently published studies have for example shown that the GT3X (without the low frequency extension) produce lower PA counts compared to the older model AM7164 [33], and applying the low frequency filter to the GT3X model results in higher PA levels (and less non-wear and sedentary time) than using the normal filter option [34].

In this study, we do not have information on whether the adolescents removed their accelerometers and if so, the exact time periods for which the monitor was not worn. Hence, we are unable to obtain “the real answer” to whether a period without recorded activity is reflecting either sedentary time or true non-wear time. Other studies have suggested that the collection of information on non-wear time in adolescents using non-wear time activity diaries can improve the ability to accurately measure PA [35]. Nonetheless, in a newly published study, in adults, the authors concluded that a logbook to record actual accelerometer time did not add any accuracy to the estimates from the accelerometer data, and furthermore created extra burdens for participants and researchers [36].

### Periods of non-wear

Finally, we analysed the distribution of the number of daily non-wear periods detected in the dataset using differing non-wear setting. As expected the number of non-wear periods was significantly reduced when lengthening the definition for non-wear time. This analysis can be used as part of the procedure in deciding on an appropriate non-wear time definition with the question being: what is a reasonable number of times that an adolescent will actually remove the accelerometer? We have not been able to find any guidelines in the literature on reasonable number of non-wear periods, but one can ask if it is likely that the accelerometer would be removed up to 17 times during the day (from 6 am to 10 pm) as was the case when analysing data with the ten minutes of non-wear time criterion. Based on our findings, we recommend future studies to critically examine the frequency of non-wear periods when applying various non-wear criteria. A study by King et al. [37] address this issue in a sample of adult bariatric surgery candidates wearing a Stepwatch Activity Monitor (worn above the ankle). The different population and use of another monitor make comparison to our study difficult. However, the authors conclude, similar to our findings, that identification of number of non-wear periods should be used as a method for deciding on an appropriate non-wear setting. We have not been able to find other studies dealing with the number of daily non-wear periods in children and adolescents.

### Limitations

In the analyses of non-wear time durations it was necessary to define valid wear time (fixing the hours/day and number of days). We chose 5 days of 10 hours, based on other studies that have recommended at least 10 hours of measurement during a day and 4/5 days of measurement as valid [6,16,17,38]. It is however, possible that the differential effect we found with regards to weight status and age would be different using a less stringent requirement for valid data, for example 3 days and 8 hours.

The use of the GT3X with the normal filter option as done in this study can potentially result in lower PA estimates and thus more sedentary time, which is important to be aware of when comparing results from this study with studies using the low frequency filter to the GT3X.

Furthermore, there are other non-wear algorithms than the ones we have used in this study, for example with regards to different “allowance settings” that could also give different results.

In this study we did not differentiate between weekdays versus weekend days. A growing body of literature has started to use lower validity criteria for weekend days compared to weekdays, and therefore it could be relevant for future studies to consider whether data reduction decisions influence the retention and PA outcomes of weekdays compared to weekend days.

Finally, the adolescents in the SPACE study were from 14 schools in the Region of Southern Denmark, all in smaller cities/non-urban areas. Therefore, it is unclear how generalisable our findings are to other geographic, ethnic, or socioeconomic groups.

## Conclusions

This study examined the impact of differing accelerometer wear time criteria in a large sample of young adolescents (11–14 years old) and found that number of participants included in the analysis and their average PA level varied based on the accelerometer wear time settings. Furthermore, overweight and older adolescents were more likely to be excluded from analyses because of missing accelerometer data, and overweight and older adolescents were more likely to be excluded when using short non-wear periods. Among adolescents with valid data there was a relative difference in non-wear time between overweight and normal weight and younger and older adolescents depending on the non-wear settings used.

Given the impact that data reduction procedures have on participants included in the analysis, on overall PA counts, and on sedentary estimates, it is important to clearly describe the data reduction choices for comparability of findings across studies.

Also, we would like to state the fact that there needs to be some consensus in the area. For better or worse, one protocol should be adopted as the standard. At present, each study is making it up as they go which doesn’t allow for comparability but also introduces different levels of error and misclassification, and this is not an ideal situation.

Future research should be dedicated to investigate how the accelerometer data reduction process can be optimized, especially with regards to definition of non-wear time. In thinking of the future, an ideal solution would be to develop a small and convenient accelerometer that could be attached to the participant’s body at all time during data collection, and which was not possible for the participant to remove during data collection. This would eliminate any discussion on whether recording of zero counts was sedentary time or true non-wear time.

## Competing interests

The authors declare that they have no competing interests.

## Authors’ contributions

MT developed the data collection protocol, coordinated the data collection, conducted the statistical analyses and drafted the manuscript. PLK contributed to the design of the analyses, conducted the statistical analyses and revised the manuscript. MO and SD contributed to the design of the analyses and revised the manuscript. LBC developed the data collection protocol, coordinated the data collection and revised the manuscript. EB assisted in the statistical analyses and revised the manuscript. JCB developed the accelerometer software programme used for data processing and helped with the interpretation of results. JT was the principal investigator of the SPACE study. All authors read and approved the final manuscript.
